# Viscoelastic properties of sweet potato complementary porridges as influenced by endogenous amylases

**DOI:** 10.1002/fsn3.492

**Published:** 2017-07-16

**Authors:** Agnes Nabubuya, Agnes Namutebi, Yusuf Byaruhanga, Reidar B. Schuller, Judith Narvhus, Trude Wicklund

**Affiliations:** ^1^ Department of Chemistry, Biotechnology and Food Science Norwegian University of Life Sciences Aas Norway; ^2^ Department of Food Technology and Nutrition School of Food Technology, Nutrition and Bio‐engineering Makerere University Kampala Uganda

**Keywords:** complementary porridge, endogenous amylases, phase angle, sweet potato, viscosity

## Abstract

Sweet potato (*Ipomoea batatas* L.) roots contain amylolytic enzymes, which hydrolyze starch thus having the potential to affect the viscosity of sweet potato porridges provided the appropriate working conditions for the enzymes are attained. In this study, the effect of sweet potato variety, postharvest handling conditions, freshly harvested and room/ambient stored roots (3 weeks), and slurry solids content on the viscoelastic properties of complementary porridges prepared using amylase enzyme activation technique were investigated. Five temperatures (55°C, 65°C, 70°C, 75°C, and 80°C) were used to activate sweet potato amylases and the optimum temperature was found to be 75°C. Stored sweet potato roots had higher soluble solids (⁰Brix) content in the pastes compared to fresh roots. In all samples, activation of amylases at 75°C caused changes in the viscoelastic parameters: phase angle (tan δ) and complex viscosity (η*). Postharvest handling conditions and slurry solids content significantly affected the viscoelastic properties of the porridges with flours from stored roots yielding viscous (liquid‐like) porridges and fresh roots producing elastic (solid‐like) porridges. Increase in slurry solids content caused reduction in the phase angle values and increase in the viscosity of the sweet potato porridges. The viscosity of the porridges decreased with storage of sweet potato roots. These results provide a possibility for exploiting sweet potato endogenous amylases in the preparation of complementary porridges with both drinkable viscosities and appropriate energy and nutrient densities for children with varying energy needs.

## 1 INTRODUCTION

Complementary foods in many developing countries are prepared from locally available sources, which are mainly starchy staples, especially cereals like maize, millet, sorghum, and rice (Kikafunda, Abenakyo, & Lukwago, 2006; Mosha, Loswai, & Tetens, 2000; Sanni, Onilude, & Ibidapo, 1999). The foods are provided in form of porridges, which usually have high viscosity due to binding of large amounts of water by the starchy structure. The porridges are too thick for young children (6–24 months of age) to consume and therefore have to be diluted in order to attain drinkable viscosity, 1,000–3,000 cP, which is appropriate for children (Kikafunda, Walter, & Abeyasekera, 1997; Cameron, Heather, & Dunn, 2009). Dilution of porridges allows the use of flour concentration of only 5–10% (Songré‐Ouattara, 2010). The resultant porridges are, however, low in energy density (0.2–0.3 kcal/g) instead of the recommended 0.7–0.8 kcal g^−1^ (Njongmeta, Ejoh, Mbofung, & Verhoef, 2003; Rombo, Taylor, & Minnaar, 2001). The energy density of the porridges can be enhanced through increase of porridge solids content, up to 20% (Nout & Ngoddy, 1997; Songré‐Ouattara, 2010; Rombo et al., 2001). However, the resultant porridges are very thick due to enormous starch content making the porridge unsuitable for young children (Nout & Ngoddy, 1997). A number of methods have been employed in an attempt to modify the behavior of starch during the heating process in order to deal with the issue of paste bulkiness. These include precooking, extrusion, fermenting, malting, and use of enzymes. Some of these methods have registered some level of success (Amankwa, Barimah, Nnaji, & Addai, 2009; Kikafunda et al., 2006; Potter & Hotchloss, 1995).

Sweet potato roots present another option besides cereals, for use as complementary foods. Sweet potato roots, as staple foods in most developing countries (Adenuga, 2010), complement the energy density of complementary foods. Besides the high starch content (60–75% DM) and considerable quantities of beta carotene found in some varieties (Adenuga, 2010; Bovell‐Benjamin, Gichuhi, & Abdall, 2004; Haskell et al. 2004), sweet potato roots also contains endogenous amylases. Sweet potato amylases can influence the roots' starch content especially in storage and during processing (Morrison, Pressey, & Kays, 1993; Nabubuya, Namuteb, Byaruhanga, Narvhus, Stenstrøm, et al., 2012; Prasanna 2005) due to their hydrolytic effect (Picha, 1985). The potential of sweet potato roots in complementary feeding has not been fully tapped into. The roots are usually steamed, pureed, and served to young children or milled into flour for use in porridge preparation (Wireko‐Manu, Ellis, & Oduro, 2010). When used in the preparation of porridges, the resultant porridges are very thick, making it difficult for young children to consume (Rombo et al., 2001). It has been reported that sweet potato amylase activity varies with variety and that it increases during storage (Morrison et al. 1993; Nabubuya, Namuteb, Byaruhanga, Narvhus, Stenstrøm, et al., 2012). The increase in amylase activity during storage leads to hydrolysis of starch, thereby reducing the quantity of native starch (Rocha, Carneiro, & Franco, 2010), given the right conditions (activated). This coupled with increased reducing sugar content leads to reduction in the viscosity of resultant pastes (Prasanna, 2005. It is, however, not clear to what extent the variations in the sweet potato root amylase activity influence the viscoelastic properties and hence energy densities of the resulting porridges, given optimum processing conditions. This study aimed at: (1) optimizing the activity of sweet potato endogenous amylases and (2) evaluating the effect of sweet potato variety, postharvest handling conditions, and solids content on the viscoelastic properties of sweet potato weaning porridges prepared using amylase enzyme activation technique.

## 2 MATERIALS AND METHODS

### 2.1 Sweet potato materials and treatments

Two sweet potato varieties (Kakamega and NASPOT 10) were used in this study and these were obtained from a farmer in Luwero district in central Uganda. The varieties were chosen based on their variations in amylase activity, both α and β amylases and starch content (Nabubuya, Namuteb, Byaruhanga, Narvhus, Stenstrøm, et al., 2012). Freshly harvested roots and stored roots (3 weeks at ambient conditions; 23–26°C and 75–80% relative humidity) were the sample Treatments. Sweet potato roots were washed under running water, peeled, sliced into small and thin pieces, and oven dried at 45°C for 20 hr. The dried sweet potato pieces were milled into flour (250 μm mesh) using a WonderMill laboratory mill (model 70, Korea). The flour was then stored in airtight containers and kept at 4°C.

### 2.2 Optimizing sweet potato amylases

Sweet potato (Kakamega variety) slurries (30% solids content w/v) were heated at various temperatures (55°C, 65°C, 70°C, 75°C, and 80°C) in a water bath. The slurries were then held at specified temperatures and change in soluble solids content (⁰Brix) was measured at 10‐min intervals for 140 min using a portable refractometer (model C‐3, 5901003, FG‐104, Barcelona). The optimum time required to attain the peak ⁰Brix at each given temperature was recorded. Sweet potato slurries from fresh and stored NASPOT 10 and Kakamega varieties (30% solids content w/v) were heated and held at 75°C for 140 min, during which period changes in soluble solids (⁰Brix) were measured at 10‐min intervals.

### 2.3 Viscoelastic measurements

The viscoelastic properties of the sweet potato porridges were measured using a Physica USD200 rheometer (Paar Physica, Anton Paar, Germany) fitted with a stirrer (FL100/Q0) and a peltier element (TEZ 150P). The porridges were prepared in the rheometer cup by mixing 9, 10.5, and 12 g of flour in 21, 19.5, and 18 mL of distilled water (30%, 35%, and 40% slurry solids content, w/v). The temperature of the slurries was raised to 75°C at a rate of 3.5°C min^−1^ and held at 75°C for 60 min to facilitate hydrolysis of starch by amylases (Mukisa et al., 2012). It was then raised to 90°C at a rate of 3°C min^−1^ and temperature maintained for 15 min and subsequently cooled to 40°C at 5°C min^−1^. Shear viscosity was measured only at the beginning and end of the preparation cycle at a constant rotational speed of 400 rpm. Nondestructive stress dynamic oscillatory measurements were used to assess the structural changes in the viscoelastic gel formed upon starch gelatinization. The viscoelastic properties measured at 10 Hz frequency and 0.1% strain were the phase angle (Tan δ), which represents the viscoelastic behavior of a food material and ranges from 0° to 90° for viscoelastic and viscous materials, respectively, and complex viscosity (η*), which is a frequency‐dependent viscosity function determined during forced harmonic oscillation of shear stress (Tabilo‐Munizaga & Barbosa‐Cánovas, 2005).

### 2.4 Statistical analysis

The data were subjected to ANOVA (general linear model) using Minitab software (Minitab, Inc., USA) version 16 and the means were separated using Tukey's test. Significance was accepted at *p* <.05 The analyses were done in triplicate and the results are presented as means with respective standard deviations.

## 3 RESULTS AND DISCUSSION

### 3.1 Optimization of sweet potato amylases

**Figure 1:**
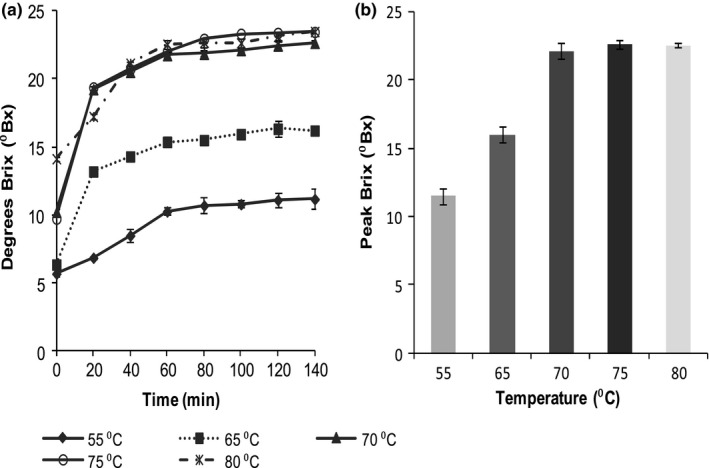
Saccharification of sweet potato slurries of Kakamega variety at different temperatures. (a) Changes in soluble solids (⁰Brix), (b) peak ⁰Brix

The soluble solids in the sweet potato pastes increased rapidly and reached maximum levels after which time there was no noticeable change (Figure 1a). The change in ⁰Brix was lowest in slurries held at 55°C (5.7 ± 0.2 to 11.2 ± 0.76 ⁰Brix) and highest in slurries held at 75°C (9.7 ± 0.21–23.4 ± 0.38 ⁰Brix). Temperature had a significant effect (*p* <.05) on the soluble solids content of sweet potato pastes. It was also observed that time significantly influenced (*p* <.05) the variation in ⁰Brix. The time to attain the peak ⁰Brix in the pastes ranged from 60 to 80 min beyond which there was no observable change (Figure 1a).

These results showed a rapid change in soluble solids during the first 20 min with no major changes thereafter. These results are consistent with those by other researchers who reported a linear increase in soluble solids over temperature ranges 30–80°C (Mcardle & Bouwkamp, 1986). The results from this study, showing very minimal changes in soluble solids at temperature ranges from 70 to 80°C, are in agreement with Nabubuya, Namuteb, Byaruhanga, Narvhus, Stenstrøm, et al. (2012) who observed that the activity of amylases in sweet potato flours is highest at around 80°C, beyond which temperature the enzymes are denatured.

The highest peak ⁰Brix in this study was obtained after holding the sweet potato slurries at 75°C and 80°C (23.43 ± 0.38 and 23.46 ± 0.15 ⁰Brix) and the lowest was 11.16 ± 0.75, obtained on subjection to 55°C (Figure 1b).

### 3.2 Changes in soluble solids with respect to variety and postharvest handling

**Figure 2:**
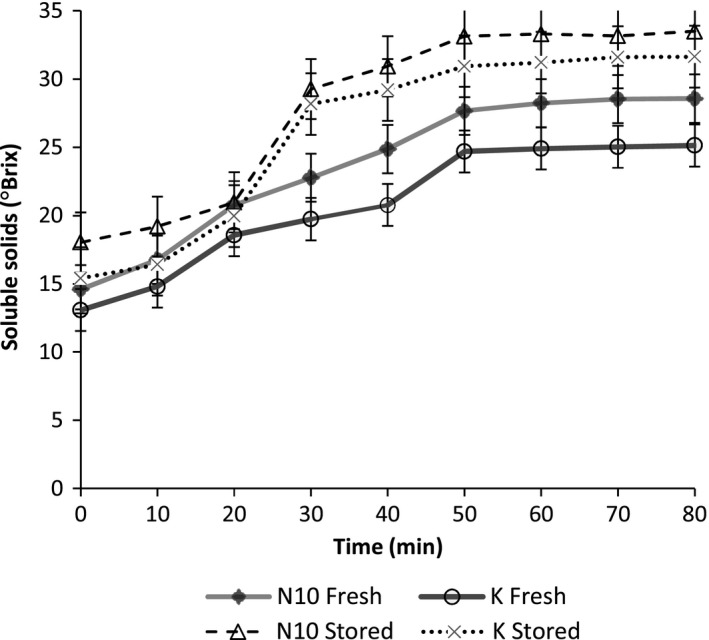
Effect of variety and postharvest handling conditions on sweet potato paste soluble solids during heating at 75°C

The soluble solids in slurries from the two sweet potato varieties increased, reached maximum, and remained constant thereafter (Figure 2). There was significant increase (*p* <.05) in the ⁰Brix during heating (18.0 ± 0.75–33.5 ± 0.53). Pastes from NASPOT 10 (N10) variety had significantly higher ⁰Brix (*p* <.05) than those from Kakamega (K). Additionally, pastes from stored roots had significantly higher ⁰Brix compared to those from freshly harvested roots (31.6 ± 0.15 and 25.1 ± 0.15, respectively) (Fig 2). The differences observed in soluble solids content between the sweet potato varieties are attributable to the differences in the activity of amylase in these varieties (Nabubuya, Namuteb, Byaruhanga, Narvhus, & Wicklund, 2012). With respect to postharvest handling, samples from stored roots contained significantly (*p* <.05) more soluble solids than the fresh ones. This is attributed to the increase in reducing sugars as a result of increased hydrolytic effect of endogenous amylases during the storage of sweet potato roots, hence breaking down native starch as the sweet potato roots prepare for sprouting (Takahata, Noda, & Sato, 1995; Morrison et al., 1993). These results are in agreement with those in literature regarding varietal differences and effects of storage (Hoover & Harmon, 1967).

### 3.3 Variation in viscoelastic properties of sweet potato pastes

**Figure 3:**
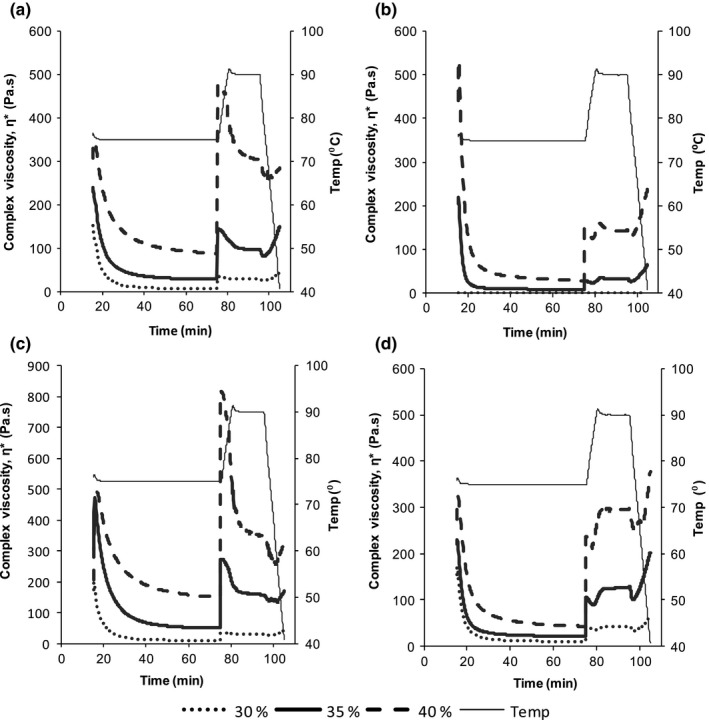
Effect of variety, postharvest handling conditions, and slurry solids content on complex viscosity (η*) of sweet potato porridges. (a) Fresh NASPOT 10, (b) stored NASPOT 10, (c) fresh Kakamega, and (d) stored Kakamega

Changes in the viscoelastic properties in all the sweet potato pastes were instantaneous on attainment of 75°C, regardless of variety, postharvest handling condition, or solids content. The complex viscosity (η*) of the pastes reduced at 75°C, reached minimum levels in 20 min, and remained constant thereafter (Figure 3). When the temperature was increased to 90°C, there was an increase in complex viscosity to maximum values, which remained constant on holding the pastes at 90°C before increasing further, on lowering the temperature to 40°C. The results revealed that postharvest handling conditions, and slurry solids content had significant effects (*p* <.05) on the porridge complex viscosity values. It was observed that the complex viscosity values were higher in pastes prepared from freshly harvested roots compared to cured roots, irrespective of the variety. It was also observed that slurries containing 30% and 40% flour solids content from both fresh and stored roots yielded porridges with the lowest and highest final complex viscosity (45.2 ± 10 and 284 ± 94 Pa.s, 65 ± 39.7 and 239 ± 54.7 Pa.s, respectively, Figure 3a and 3b).

**Figure 4:**
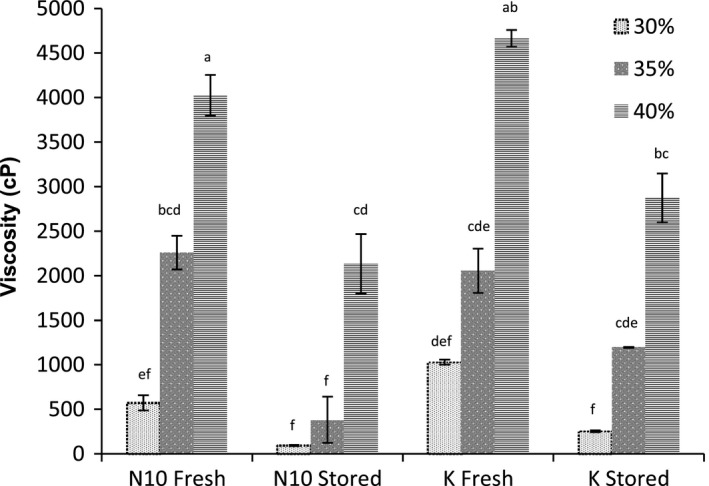
Effect of variety, postharvest handling conditions, and slurry solids content on the final shear viscosity of sweet potato porridges. Values are mean ± standard deviation (*n* = 3); different lower case letters indicate significant differences at *p* <.05

Results obtained during constant rotation at the end of the cooling cycle (40°C) revealed that slurry solids content significantly (*p* <.05) affected the final shear viscosity of the sweet potato porridges (Figure 4). The shear viscosity increased with increase in slurry solids content (1,030 ± 28 cP, 2,055 ± 249 cP, and 4,662 ± 94 cP for 30%, 35%, and 40%, respectively). It was observed that flours from stored roots yielded porridges with very low viscosities (382 ± 59 and 1,196 ± 4.1 cP for N10 and K, respectively) compared to porridges from freshly harvested roots (2,258 ± 189 and 2,055 ± 248 cP, respectively; Figure 4).

**Figure 5:**
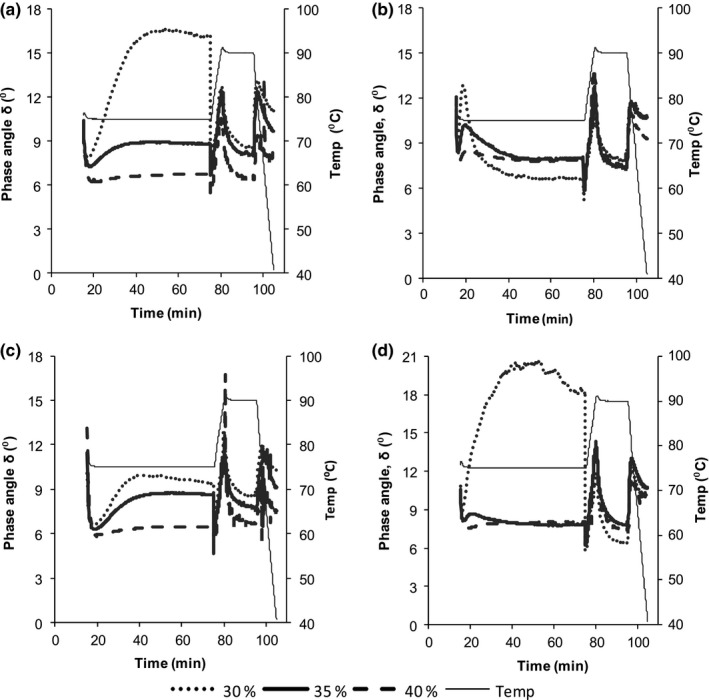
Effect of variety, postharvest handling conditions, and slurry solids content on the phase angle (tan δ) of sweet potato porridges. (a) Fresh NASPOT 10, (b) stored NASPOT 10, (c) fresh Kakamega, and (d) stored Kakamega

The phase angle, tan δ (describing the viscoelastic behavior of the pastes), increased (samples became liquid like) at 75°C, then dropped on increasing temperature to 90°C (became solid like), where it increased again and dropped gradually when samples were being held at 90°C (Figure 5). The phase angle finally decreased during cooling to 40°C. Postharvest handling conditions and slurry solids content significantly (*p* <.05) affected the phase angle of the samples. Storage of sweet potatoes increased the phase angle of the porridges, hence more liquid‐like products on cooling compared to use of fresh sweet potatoes (10.7 ± 0.1 and 9.2 ± 0.12, respectively) for Kakamega at 35% solids content (Figure 5c and d). The slurry solids content also significantly influenced the phase angle of the porridges. Values were higher in samples with low solids content (11 ± 0.49, 9.7 ± 0.32, and 7.9 ± 0.52) for 30%, 35%, and 40%, respectively (Figure 5a).

The decrease observed in complex viscosity during saccharification (at 75°C) in all samples coincided with increase in phase angle values and soluble solid content (⁰Brix). This is attributable to increase in the activity of endogenous amylases on the starch contained in the sweet potato slurries and the increase in reducing sugars like glucose, sucrose, and fructose (Nebesny, 1993). Sweet potato endogenous amylases (α and β) hydrolyze the glycosidic bonds in the gelatinized starch during saccharification, liberating maltodextrins and maltose, respectively (van der Maarel, van der Veen, & Uitdehaag, 2002). It has however been reported that α‐amylases are the major players in controlling viscosity due to their higher ability to hydrolyze starch, and that β‐amylases, while causing significant change, do not have as much effect as that caused by the former (Sarikaya, Higasa, Adachi, & Mikami, 2000). The liquefaction effect of the amylases in the sweet potatoes led to the decrease observed in complex viscosity and increase in phase angle values. A phase angle of 90° indicates that the product is totally viscous, whereas values closer to 0° indicate an elastic product.

The increase in complex viscosity during cooling also coincided with decrease in phase angle values in all samples. This is associated with amylose chain retrogradation (Matalanis, Campanella, & Hamaker, 2009). The viscosity of porridges/gruels tends to increase as the porridges cool irrespective of the starch source. The increase in porridge viscosity with slurry solids content is consistent with Kikafunda et al. (1997) and Mouquet and Trèche (2001) who reported similar results in complementary porridges made from maize flour. The final phase angle values of the porridges in this study ranged from 7.5° to 11° indicating that the sweet potato porridges had predominately elastic rather than viscous properties and can therefore be classified as weak gels (Tabilo‐Munizaga & Barbosa‐Cánovas, 2005).

The differences observed in the final viscosities and phase angle values of sweet potato pastes from fresh and stored roots could be attributable to the variation in the quantities of native starch and sugars found in these roots. Fresh sweet potato roots contain mostly native starch, while the native starch in the stored roots is reduced in quantity and occurs alongside starch with low molecular weight (Noda, 2004) and increased levels of sugars, particularly reducing sugars (Morrison et al., 1993; Zhanga et al., 2002). Therefore, the differences in viscoelastic properties observed in this study is largely due to the reduction in swelling power of the starch from stored roots caused by the hydrolytic effect of endogenous amylases as compared to the starch from fresh roots which has full swelling potential during pasting (Adebowale & Lawal, 2003; Morrison et al., 1993; Noda, 2004). Nabubuya, Namuteb, Byaruhanga, Narvhus, Stenstrøm, et al. (2012) in a study comparing fresh and stored sweet potato roots also observed that the activity of endogenous amylases was higher in stored than in fresh roots. This implies that pastes obtained by use of flours from fresh sweet potato roots are more firm and elastic in nature, unlike those prepared from flours obtained from stored roots, which are more viscous in nature.

Viscosity is a very important characteristic of weaning foods that has influence on both the quantity of food consumed by a child and the energy intake (Kikafunda et al., 1997; Mosha & Svanberg, 1990). A desirable viscosity is that which facilitates easy consumption while maintaining adequate solids content. Extensive swelling of starch granules during the heating process has been reported to limit the solids content of these porridges to 5–10% (Rombo et al., 2001), hence leading to porridges with low energy density although having acceptable consistency (2,000–3,000 cP). The energy density of the porridges can be improved by increasing the flour content up to a minimum of 20% (Rombo et al., 2001). Results from this study revealed that weaning porridges with increased solids content, acceptable consistency (2,000–3,000 cP), and increased energy density can be obtained from sweet potato flour through saccharification of pastes at 75°C for 10–30 min.

Sweet potato varieties vary in starch content and amylase activity (Nabubuya, Namuteb, Byaruhanga, Narvhus, & Wicklund, 2012), implying that the swelling ability for their pastes vary. In this study, it was possible to make porridges containing 35% slurry solids content from flours of both fresh NASPOT 10 and Kakamega with final viscosities of 2,258 and 2,055 cP, respectively. Porridges with 40% slurry solids content were obtained from flours of stored sweet potatoes with final viscosities of 2,133 and 2,871 cP for NASPOT 10 and Kakamega, respectively. Irrespective of the postharvest handling conditions the roots were subjected to, the porridges obtained using the 35% and 40% slurry solids content contained energy densities of 1.16 and 1.33 kcal mL^−1^, respectively. These results imply that if the sweet potato slurries are subjected to the right processing conditions (75°C), the amylases in sweet potato roots irrespective of variety are activated and hydrolyze the starch to simpler sugars hence reducing the viscosity of resulting pastes. This therefore facilitates the utilization of increased slurry solids in porridge preparation, thereby increasing the energy and nutrient density of resultant complementary porridges.

## 4 CONCLUSIONS

This study confirms that endogenous amylases in sweet potato flour can be manipulated by heating at 75°C for 10–30 min to cause reduction in the viscosity of sweet potato pastes and facilitate increase in paste solids content hence increasing the energy density of the resulting porridges. The fact that porridges from freshly harvested sweet potatoes at 35% slurry solids content yielded porridges with acceptable viscosity (2,000–3,000 cP) along with 40% slurry solids content for stored roots observed in this study points to the fact that it is cheaper to exploit the use of fresh roots rather than store roots for 3 weeks to attain the same effect in terms of porridge viscosity. These results provide a possibility for use of amylases in sweet potatoes in the production of porridges for children with varying energy needs. Although saccharification led to porridges with desirable viscosity and increased energy density, it is important to mention that the caloric content of these diets was slightly above the recommended values for children still consuming breast milk. It is therefore advisable to reduce the amounts provided per feeding in order to avoid instances of over nutrition.

## CONFLICT OF INTEREST

Authors declare that there is no conflict of interest.
